# Advanced smart biomaterials and constructs for hard tissue engineering and regeneration

**DOI:** 10.1038/s41413-018-0032-9

**Published:** 2018-10-22

**Authors:** Ke Zhang, Suping Wang, Chenchen Zhou, Lei Cheng, Xianling Gao, Xianju Xie, Jirun Sun, Haohao Wang, Michael D. Weir, Mark A. Reynolds, Ning Zhang, Yuxing Bai, Hockin H. K. Xu

**Affiliations:** 1 Department of Orthodontics, School of Stomatology, Capital Medical University, Beijing, China; 2 Department of Advanced Oral Sciences and Therapeutics, University of Maryland Dental School, Baltimore, MD USA; 3 State Key Laboratory of Oral Diseases & National Clinical Research Center for Oral Diseases & Deptartment of Cariology and Endodonics West China Hospital of Stomatology, Sichuan University, Chengdu, China; 4 Department of Operative Dentistry and Endodontics, Guanghua School of Stomatology, Sun Yat-sen University, Guangdong Provincial Key Laboratory of Stomatology, Guangzhou, China; 5 Volpe Research Center, American Dental Association Foundation, National Institute of Standards and Technology, Gaithersburg, MD USA; 6 Center for Stem Cell Biology & Regenerative Medicine, University of Maryland School of Medicine, Baltimore, MD USA; 7 Marlene and Stewart Greenebaum Cancer Center, University of Maryland School of Medicine, Baltimore, MD USA

## Abstract

Hard tissue repair and regeneration cost hundreds of billions of dollars annually worldwide, and the need has substantially increased as the population has aged. Hard tissues include bone and tooth structures that contain calcium phosphate minerals. Smart biomaterial-based tissue engineering and regenerative medicine methods have the exciting potential to meet this urgent need. Smart biomaterials and constructs refer to biomaterials and constructs that possess instructive/inductive or triggering/stimulating effects on cells and tissues by engineering the material’s responsiveness to internal or external stimuli or have intelligently tailored properties and functions that can promote tissue repair and regeneration. The smart material-based approaches include smart scaffolds and stem cell constructs for bone tissue engineering; smart drug delivery systems to enhance bone regeneration; smart dental resins that respond to pH to protect tooth structures; smart pH-sensitive dental materials to selectively inhibit acid-producing bacteria; smart polymers to modulate biofilm species away from a pathogenic composition and shift towards a healthy composition; and smart materials to suppress biofilms and avoid drug resistance. These smart biomaterials can not only deliver and guide stem cells to improve tissue regeneration and deliver drugs and bioactive agents with spatially and temporarily controlled releases but can also modulate/suppress biofilms and combat infections in wound sites. The new generation of smart biomaterials provides exciting potential and is a promising opportunity to substantially enhance hard tissue engineering and regenerative medicine efficacy.

## Bone repair: a new generation of smart biomaterials

Smart biomaterials that are able to instruct bone repair can overcome some of the shortcomings of bone grafting and meet the growing need for hard tissue regeneration in ageing populations. Hockin Xu at the University of Maryland in Baltimore, United States, and colleagues review recent advances in the development of smart biomaterials for repairing and regenerating damaged bones and teeth. They highlight scaffolds that closely mimic natural bone tissue and immunomodulatory biomaterials that can prevent infection and promote cell survival for guiding and enhancing bone regeneration. Furthermore, scaffolds with shape-memory capability and materials that provide tailored spatio-temporal delivery of drugs or bioactive agents in response to internal or external stimuli, hold great promise not only for bone tissue engineering but also for dentistry.

## Introduction

In general, smart (or intelligent) biomaterials and constructs refer to those that: (1) possess instructive/inductive or triggering/stimulating effects on cells and tissues by engineering the material’s responsiveness to internal or external stimuli, such as pH, temperature, ionic strength and magnetism, to promote damaged tissue repair and regeneration; or (2) have intelligently tailored individual properties and controlled functions to actively participate in tissue regeneration in a valuable way.^1–4^ This article reviews recent developments in smart biomaterials and constructs for hard tissue repair and regeneration. Hard tissues include bone and tooth enamel, dentin and cementum. Hard tissues are also termed calcified tissues, as they contain calcium phosphate minerals. The need for hard tissue repair and regeneration has substantially increased as the world population ages.^5,6^ Bone fractures, defects and non-unions are a significant worldwide problem.^7,8^ The annual healthcare costs plus lost wages for individuals in the United States with musculoskeletal diseases reached $849 billion in 2004 or 7.7% of the gross domestic product.^8^ This cost is rapidly increasing as the population ages. Although autografts are the gold standard, the risks of donor site morbidity and limited availability restrict their applications. Allografts are impeded by potential infection and a high non-union rate with host tissues. Therefore, smart biomaterials and smart tissue engineering constructs provide immense potential as an exciting alternative to autogenous bone grafts.^5,6,9,10^

Regarding the other hard tissue, tooth caries is the most prevalent disease in humans. Dental caries creates substantial public healthcare burdens and affects oral and general health and quality of life.^11,12^ In the US, more than 200 million tooth cavity restorations are placed annually, costing $46 billion in 2005.^12^ The need is rapidly increasing with increases in the life expectancy and tooth retention rates in seniors.^13^ Caries is caused by acidogenic bacteria fermenting carbohydrates to produce acids, which leads to mineral loss.^14^ Even after a tooth cavity is restored, the restoration often fails over time, mainly due to secondary (recurrent) caries.^15^ Moreover, the replacement of failed restorations accounts for more than half of all restorations placed.^16^ Therefore, there is an immense need to develop a new generation of smart dental restorations to reduce and eliminate caries. This article covers both bone and teeth because many of the smart biomaterials and smart constructs are applicable to both types of hard tissues.

## Smart scaffold constructs with stem cells for bone tissue engineering

Scaffolds, cells and growth factors are the three basic elements of bone tissue engineering.^17^ Scaffolds are not only a substitute for the extracellular matrix (ECM) but can also serve as the delivery vehicle for cells and the carrier for growth factors.^18^ Scaffolds affect seeded cells, including cell attachment, migration and proliferation, thus affecting the efficacy of regenerative medicine.^19^ Smart scaffolds have been designed with the incorporation of bioactive molecules and nanoparticles and the use of tailored modifications of the physical and chemical properties of the scaffolds.^20,21^ They can improve the interactions with cells by enhancing the osteogenic differentiation for bone repair and responding better to the surrounding host environment.^3^ Scaffolds were composed of natural or synthetic materials or their combinations, and their advantages included that they could incorporate bone progenitor cells and growth factors to display osteoconductive and osteoinductive potentials and help replace and repair bone defects. The typical deficiencies of traditional scaffolds included material-related infections, mechanical failures and adverse immunogenic reactions with the host. Recent studies have developed novel smart scaffold constructs to improve the tissue regeneration efficacy.

### Biomimetic and bionic smart scaffolds

An important class of smart materials is referred to as biomimetic smart materials. Their development is based on the biological inspiration of the structure, function and formation of biological materials.^22^ Engineering multifunctional and adaptive cellular microenvironments is imperative in developing native tissue-like biomaterials. The cell–biomaterial interface is a complex and dynamic microenvironment and is important in tissue regeneration.^23^ Stem cells in contact with the scaffold can sense different properties, such as stiffness and nanostructure, and make the appropriate responses, thus enabling smart scaffolds to induce the desired cell responses. For example, previous studies have indicated that innate immune cells, particularly macrophages, could undergo phenotypical changes after being challenged by the tailored material cues, which could facilitate tissue regeneration.^24^

Recently, smart artificial bone scaffolds were prepared to mimic the composition and structural characteristics of natural bone using the principles of biomimetics, nano-assembly technology and additive manufacturing techniques.^25^ Specific molecular recognition signals, such as peptides, growth factors and genes, were immobilized on the scaffold. Biomimetic porous poly(lactide-co-glycolide) (PLGA) microspheres coupled with peptides were used to construct biomimetic environments for tissue engineering.^26^ Based on the analysis of the porous structures of trabecular bone, computer-aided porous scaffold design for tissue engineering could describe the surface morphology and pore size distribution of the bone microstructure.^27^ Thus, the smart scaffold could closely mimic the true features of the skeleton and follow the complexity of the natural bone tissue structures in vivo, thereby holding substantial potential as guidance templates for cells to enhance bone regeneration.

### Immune-sensitive smart scaffolds

However, scaffolds with poor biocompatibility can trigger aggressive foreign-body reactions in vivo. To avoid or lessen the potential immunological response between the host immune system and foreign scaffolds, it is important to develop smart immunomodulatory biomaterials that are capable of directing the host response towards tolerance of the foreign scaffolds or regulate immunological microenvironments to promote cell survival. The immune system is the first responder of the host and plays a critical role in responding to tissue trauma and the implantation of biomaterials.^28^ The immune system consists of the innate and adaptive immune systems, which play key roles in combating disease and infection. Immune-sensitive smart scaffolds with an osteoimmunomodulatory capability could provide an osteoconductive microenvironment to enhance stem cell survival and regenerative functions.^29^

**Fig. 1:**
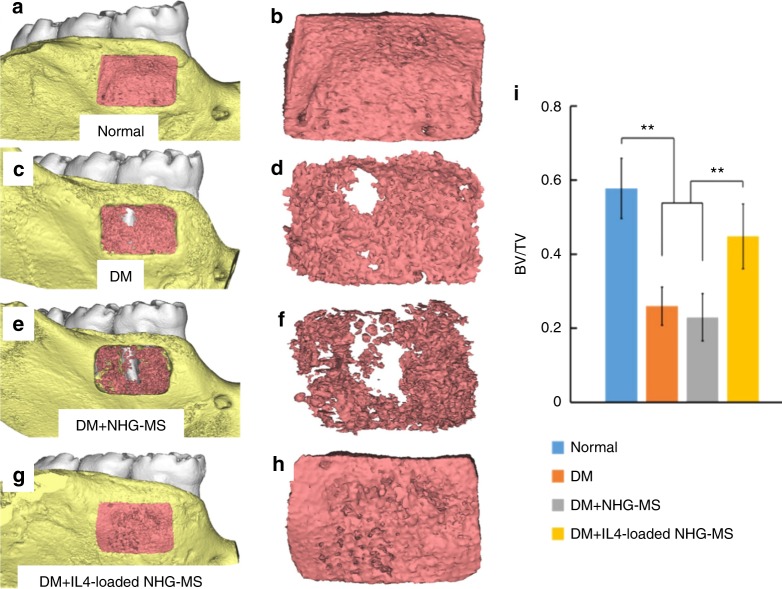
Smart immunomodulatory microspheres (NHG-MS) accelerated bone regeneration in diabetes mellitus (DM). µ-CT at 4 weeks confirmed that bone regeneration was impaired by diabetes. However, IL4-loaded NHG-MS restored the bone regeneration to the healthy non-diabetic level. The defect was filled with new bone in the healthy non-diabetic control (**a**, **b**) and the IL4-loaded NHG-MS DM (**g**, **h**). In contrast, only half of the defect was filled with new bone in the DM (**c**, **d**) and DM + NHG-MS (**e**, **f**). **i** The ratio of bone volume to total volume (BV/TV) of the non-diabetic group was twice that of the DM group. IL4-loaded NHG-MS in DM rats increased the BV/TV ratio to 0.44, twofold that of the NHG-MS group (0.23). (**P < 0.01). (Adapted from ref. ^34^, with permission.)

Surface modification of scaffolds could be beneficial for immune cell activation and infiltration.^30^ ECM-like injectable gelatin microspheres were synthesized via the self-assembly of heparin-modified gelatin nanofibres.^31–33^ Interleukin 4 (IL4) was incorporated into the scaffold by binding the domains with heparin to protect it from denaturation and degradation, which helped prolong its sustained release to modulate the macrophage polarization.^34^ The nanofibrous heparin-modified gelatin microspheres (NHG-MS) could spatiotemporally deliver the anti-inflammatory cytokine IL4 to polarize the proinflammatory M1 macrophages into an anti-inflammatory M2 phenotype, thus facilitating osteogenic differentiation and bone formation.^34^ The release of IL4 was not pH- or temperature-sensitive. However, the smart design was that the IL4 was incorporated into the nanofibrous gelatin microspheres through binding with heparin, which mimicked the binding of cytokines and growth factors with glycosaminoglycan in natural ECM. In addition, heparin had a specific binding domain with IL4. The smart binding of IL4 with heparin helped to stabilize and protect the IL4 from degradation and controlled the sustainable release of the bioactive IL4 for up to 3 weeks. Furthermore, even under diabetic conditions, the IL4-loaded immunomodulatory microspheres significantly enhanced bone regeneration, as shown in a diabetes mellitus (DM) rat mandibular periodontal defect model in Fig. 1.^34^ The micro-computed tomography (µ-CT) analysis at 4 weeks after surgery demonstrated that the IL4-loaded NHG-MS restored the close to the normal level in the presence of diabetes. The defect area was entirely occupied by new bone in the non-diabetic control group (Fig. 1a, b) and the IL4-loaded NHG-MS DM group (Fig. 1g, h). In contrast, only half of the defect was filled with new bone in the DM (Fig. 1c, d) and DM + NHG-MS groups (Fig. 1e, f). The ratio of the bone volume to total volume (BV/TV) of the IL4-loaded NHG-MS DM group (0.44) was approximately twofold that of the DM + NHG-MS group (0.23) (Fig. 1i).^34^

In addition, an amino-functionalized bioactive glass (MBG) scaffold was developed to investigate its effects on bone marrow mesenchymal stem cells (BMSCs) and macrophages.^35^ The osteogenic ability of the MBG scaffold was enhanced via amino functionalization and could coordinate BMSCs and macrophage differentiation. The osteoimmunomodulatory capability of the scaffold was endowed by the amino functionalization.^35^ Furthermore, β-tricalcium phosphate (β-TCP), with reported favourable osteoimmunomodulatory properties, was used to coat Mg scaffolds to modulate the detrimental osteoimmunomodulatory properties of Mg scaffolds.^36^ β-TCP-coated Mg scaffolds induced macrophages to switch to the extreme M2 phenotype, which caused the release of anti-inflammatory cytokines and osteoinductive molecules by the macrophages. This yielded an upregulation of bone morphogenetic protein (BMP) expression by 25-fold and enhanced the osteogenesis of BMSCs.^36^ Therefore, osteoimmunomodulatory smart biomaterials have displayed favourable osteoinductive properties. Additional efforts should be devoted to endow the bone biomaterials with favourable osteoimmunomodulatory properties to trigger the desired immune response and promote bone regeneration.

### Shape-memory smart scaffold**s**

Another class of smart scaffolds have a shape-memory capability. Shape-memory polymers (SMPs) can return from a deformed shape to their original shape by an external stimulus, such as temperature change,^37^ an electric or magnetic field,^38,39^ and light.^40^ They have received substantial attention as a result of their applications in tissue engineering.^41^ The advantages of the shape-memory behaviour are that the scaffolds can be predesigned, deformed to be conveniently implanted into bone defects via minimally invasive surgery and then expanded to adapt to an irregular bone defect.^42^ The initial implant has a small size and can be deployed in the body using minimally invasive means with the least damage to host tissues. After deployment, the implant regains a larger shape to fill the bone defect, even precisely matching irregular bone defect boundaries.^43,44^

**Fig. 2:**
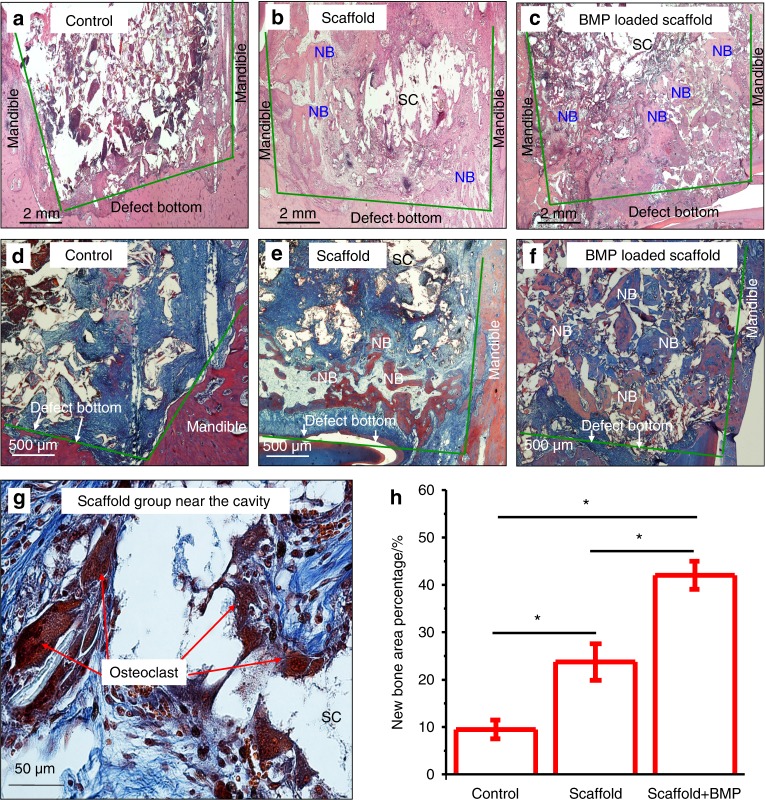
BMP2-loaded shape-memory scaffold promoted bone formation: **a**–**c** HE staining; **d**–**g** Masson staining; and **h** quantification. The defect in the control (no scaffold) was full of granulation tissues (**a**). There was more neonatal bone in defects in the scaffold group (**b**, **e**). BMP2-loaded scaffold group (**c**, **f**) had the greatest amounts of new bone and mature bone. **g** High magnification Masson staining showed many multinuclear cells. **h** BMP2-loaded scaffold group had the greatest new bone percentage based on quantitative analysis (*P < 0.05). (Adapted from ref. ^45^, with permission.)

BMP2-loaded shape-memory porous nanocomposite scaffold (SMP scaffold) that consists of chemically crosslinked poly(ε-caprolactone) and hydroxyapatite (HA) nanoparticles was fabricated for the repair of bone defects.^45^ The porous scaffold displayed shape-memory recovery from the compressed pores of 33 μm in diameter to recover its original porous shape of 160 μm in diameter, under both in vitro and in vivo conditions. In vivo µ-CT and histomorphometry results demonstrated that the BMP2-loaded SMP scaffold promoted bone regeneration in rabbit mandibular bone defects.^45^ As shown in Fig. 2, no mature bone trabecula was formed in the defect of the control group without a scaffold (Fig. 2a, d). Newly formed bone within the defect was identified in the SMP scaffold group (Fig. 2b). In contrast, nearly complete new bone and trabecula-like structures were formed in the BMP2-loaded scaffold group (Fig. 2c, f). Quantification of neonatal bone in the defect at 8 weeks showed that the BMP2-loaded scaffold group generated the highest new bone percentage compared to the other groups (Fig. 2h).^45^

Based on three-dimensional (3D)-printing technologies, four-dimensional (4D)-printed hierarchy scaffolds were created using a series of novel SMPs, demonstrating excellent biocompatibility and tunable shape-changing effects.^46^ 4D printing refers to the 3D printing of active materials, such as SMPs, along with the fourth dimension (refers to time) to have time-dependent shape transforms after printing when exposed to environmental stimuli.^46^ The smart polymeric scaffolds displayed finely tunable recovery and showed excellent attachment, proliferation and differentiation of MSCs.^46^ Furthermore, 4D shape-memory polyurethane scaffolds were also produced via 3D printing.^47^ An intrinsic mechanical stimulus provided by controlling the time-dependent morphing recovery of the scaffold was shown to significantly elongate the cells and the nuclei. Thus, the activity of cells seeded on SMP scaffolds could be effectively directed via multiple mechanical stimuli by optimally programming the cycle of shape recovery.^47^

### Electromechanical-stimulus smart scaffolds

In addition, another important class of smart materials consists of electromechanical-stimulus scaffolds. The discovery of electric fields in biological tissues has led to the development of technologies that utilize electrical stimulation for therapies.^48^ The piezoelectric effect is the ability of certain materials to generate an electric charge in response to an applied mechanical stress.^49^ Piezoelectric materials include certain crystals and ceramics, some living tissues (such as natural bone, tendon, ligaments, cartilage, skin, dentin and collagen) and certain biological macromolecules (such as proteins, nucleic acids and mucopolysaccharides).^49^ Biological electric fields in host tissues play significant roles in functions that include neuromuscular activity, glandular secretion, cell membrane function, and tissue growth and repair.^50^ Efforts were made to develop smart electrically active biomaterials and scaffolds, demonstrating potential for bone engineering by providing electrical stimulation to cells to promote tissue formation.^51^

Piezoelectric materials enhanced tissue formation by providing an electrically active microenvironment without the need for external power sources for electrical stimulation.^52,53^ Piezoelectric poly(vinylidene fluoride-trifluoroethylene) (PVDF-TrFE) was fabricated into flexible, 3D fibrous scaffolds.^54^ The scaffolds stimulated MSC differentiation and tissue formation when undergoing dynamic loading, which mimicked the physiological loading conditions in structural tissues in vivo.^54^ The piezoelectric scaffolds with dynamic compression at 1 Hz frequency with 10% deformation induced greater MSC chondrogenic differentiation than mechanical loading alone. Histological staining clearly showed chondrocyte morphology and intense proteoglycan staining, as well as collagen type II immunostaining for electrospun PVDF-TrFE after 28 days, which was not detected in the control. Similarly, MSC osteogenic differentiation was promoted on piezoelectric scaffolds, with Alkaline phosphatase activity (ALP) and mineralization fivefold those for the control.^54^ These results are consistent with another study showing that an electrospun PVDF-TrFE fibre scaffold containing zinc oxide nanoparticles promoted the adhesion and proliferation of human MSCs (hMSCs) and enhanced the blood vessel formation in a rat model.^55^

Furthermore, piezoelectric HA/barium titanate (BaTiO_3_) composite was fabricated and exposed to a dynamic loading device that simulated the force of human motion.^56^ This provided periodic loading to the piezoelectric material while co-culturing with osteoblast cells.^56^ When cyclic loading was applied on HA/BaTiO_3_, the electrical stimulation promoted osteoblast proliferation and growth, which was similar to the piezoelectric effects on human bone growth, modelling and reconstruction in vivo.^56^ Therefore, electromechanical-stimulus smart scaffolds are promising for culturing with stem cells to enhance bone repair and regeneration.

## Smart drug delivery for bone tissue engineering

In addition to the ability of smart scaffolds to interact with cells and induce the desired cell functions for tissue regeneration, scaffolds can also be used to deliver drugs. Bioactive factors, including small molecules, cytokines, peptides, proteins and genes, can be loaded in drug delivery systems (DDS).^57^ These smart systems can have controlled drug release and exert synergistic effects from multiple loaded drugs to enhance osteoblast proliferation and differentiation to promote bone regeneration.^58,59^ Several types of stimuli-responsive smart materials have been developed for drug delivery.^60,61^ In addition, strategies are being investigated to spatially and temporarily control the delivery patterns of bioactive factors to optimize bone engineering efficacy. With the help of the controlled and targeted DDSs, the drugs incorporated into the smart systems could be delivered to the exact location with the correct dosage. This approach provides the tailored spatio-temporal delivery of therapeutic agents, thereby minimizing the side effects and maximizing the therapeutic efficacy. However, these DDSs exhibited several shortcomings, including potential cytotoxicity, limited biodegradability and potential adverse immune responses, which warrant further investigation to overcome.

### Stimuli-responsive tunable DDSs

One novel strategy employs stimuli-responsive tunable DDSs. These materials can change their properties induced by a small stimulus; thus, they can deliver the required amount of drug on-demand by responding to the endogenous and/or exogenous stimulus.^62^ Examples of exogenous stimuli include an electric field, magnetic field, ultrasound, electromagnetic radiation and temperature, which may be used to turn on or off the drug release from the carriers.^63,64^ Examples of endogenous stimuli include pH, temperature, ionic environment, proteins and carbohydrates.^63,64^

Stimuli-responsive smart materials were developed that could enable real-time non-invasive or minimally invasive drug delivery and monitoring.^65^ Polymers with controlled, tunable and reversible responses to environmental stimuli were shown to be excellent candidates for drug delivery.^66^ In addition, smart hydrogel DDSs with predictable and tunable drug release and degradation rates were developed.^67,68^ A novel pH-responsive bacterial cellulose-g-poly(acrylic acidco-acrylamide) hydrogel with a highly porous morphology was developed as an oral controlled-release drug delivery carrier.^67^ The intelligent hydrogel was demonstrated to exhibit remarkable pH-responsive changes in swelling behaviour, with decreased swelling in acidic media and maximum swelling at pH 7. Thus, these hydrogels could be suitable candidates for controlled drug delivery responding to pH changes in the host physiological environments. Another poly(ethylene glycol) hydrogel was loaded with drugs by β-eliminative linkers and demonstrated tunable capability in drug release and the hydrogel erosion rate, showing good potential for applications in regenerative medicine and orthopaedic implants.^68^ In addition, farnesol-loaded nanoparticles, composed of 2-(dimethylamino)ethyl methacrylate (DMAEMA), butyl methacrylate (BMA) and 2-propylacrylic acid (PAA) (p(DMAEMA)-b-p(DMAEMA-co-BMA-co-PAA)) were synthesized with a pH-responsive drug release capability due to microenvironmental triggers.^69^ They were tuned to expedite the drug release when the local cariogenic biofilm microenvironmental pH became acidic at pH 4.5. This suggested that the tunable nanoparticle-mediated drug delivery approach could be used to deliver drugs to treat and respond to biofilm-related infections. Furthermore, a dual-responsive DDS was developed by coating electro-sensitive and thermally responsive functional macromolecules to mesoporous silica nanospheres.^70^ This system could act as a rate modulator to regulate the diffusion kinetics of the drugs loaded in the channels of the inorganic nanocarriers.^70^ The released quantities could be continuously tuned by changing the frequency of the applied external electric field under different temperatures. These smart and tunable DDSs with a flexible modulation capability of the release quantity have great potential in biomedical engineering and tissue regeneration applications.

### Smart multifunctional nanoparticle-based DDSs

Another strategy consists of multifunctional nanoparticle-based drug delivery. With the advancements in nanotechnology, various nanoparticles with dimensions of 10–200 nm are recognized as promising drug transport vehicles due to their favourable biological properties, small size and high surface area, surface chemistry and the ease with which they are taken up by cells.^71^ Typically, drugs, growth factors and genetic materials are entrapped or encapsulated in, or attached to, the nanoparticles for delivery.^72,73^ One type of nanoparticle for drug delivery included mesoporous silica nanoparticles (MSNs). MSNs had variable pore structures and large active surface areas, which enabled the attachment of different functional groups to achieve precisely controlled drug release and targeted delivery.^74^ These nanoparticles were imparted with smart features to better react to the biological environment and meet the on-demand therapeutic and diagnostic purposes of diseased and damaged tissues. Multifunctional nanomaterials were proposed to enable the simultaneous target imaging and on-demand delivery of therapeutic agents to the specific tissue site.^22^ Stimuli-responsive controlled-release MSNs were designed that responded to internal stimuli, including enzymes, pH and temperature, as well as external stimuli, including light, ultrasound and magnetic fields.^75,76^

**Fig. 3:**
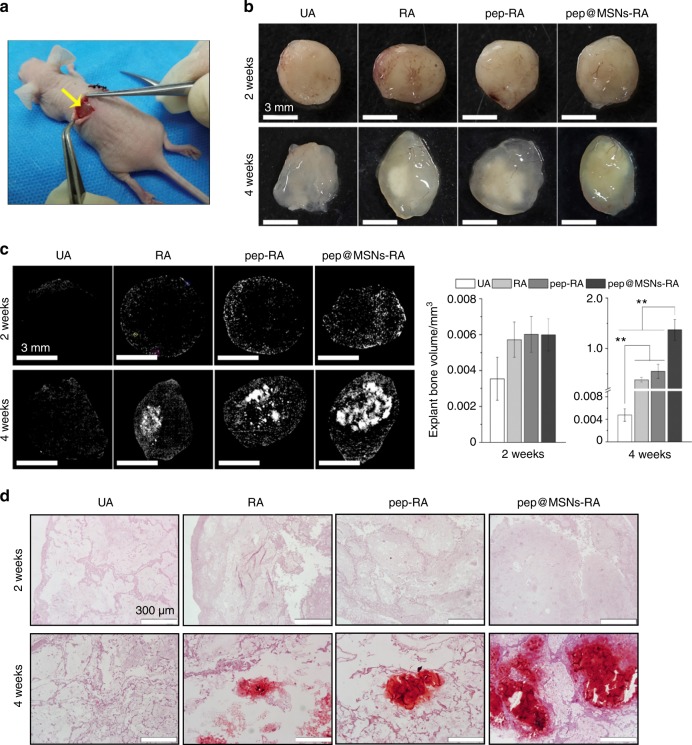
Smart dual-peptide alginate nanoscale drug delivery (pep@MSNs-RA) promoted bone mineralization. **a** Hydrogels (UA, RA, pep-RA and pep@MSNs-RA) encapsulated with hMSCs were subcutaneously implanted in nude mice. **b** hMSC-loaded gels following removal from mice at 2 and 4 weeks after surgery. **c** µ-CT reconstruction at 2 and 4 weeks. Little mineralization occurred in the groups at 2 weeks. At 4 weeks, RA, pep-RA and pep@MSNs-RA exhibited mineralization; substantially more mineralized bone tissues were identified in the pep@MSNs-RA group. **d** ARS staining. Minerals occurred in the hMSC-loaded groups (RA, pep-RA and pep@MSNs-RA), while no mineral occurred in UA (**P < 0.01). (Adapted from ref. ^77^, with permission.)

Bone-forming peptide-1 (BFP-1)-laden MSNs (pep@MSNs) were encapsulated into arginine-glycine-aspartic acid-treated alginate hydrogel (RA), which was referred to as pep@MSNs-RA.^77^ MSNs contained many uniform and homogeneous pore channels; thus, they could be applied as an excellent drug carrier for BFP-1. The smart pep@MSNs showed a sustained peptide release pattern in the appropriate growth stage of hMSCs upon demand, with the initial osteo-inducing time likely on the fourth day. A smart sequencing stimulation on the osteo-differentiation of hMSCs was achieved, in which the survivability, spreading, expansion and aggregation of hMSCs was promoted by an adhesion ligand (RGD)-modified alginate matrix at an early stage. The osteogenic factor (BFP-1) release from pep@MSNs subsequently induced the osteo-differentiation.^77^ The different functional time of BFP-1 could be regulated by changing the pep@MSN concentration in the gel. Furthermore, an in vivo test examined bone regeneration via pep@MSNs-RA encapsulated with hMSCs, with hMSC-loaded untreated hydrogel as the control (UA), for subcutaneous implantation in nude mice.^77^ In Fig. 3a, various gels (including UA, RA, pep-RA and pep@MSNs-RA) encapsulated with hMSCs were subcutaneously implanted into nude mice. In Fig. 3b, different hMSC-loaded gels following removal from mice at 2 and 4 weeks after surgery are shown. The µ-CT analysis results in Fig. 3c shows that there was little mineralization in the groups at 2 weeks. At 4 weeks, mineralized bone was identified in pep@MSNs-RA, which was approximately 300-fold higher than the control group by quantitative bone volume analysis.^77^ Alizarin Red-S staining in Fig. 3d supported the µ-CT analysis.^77^ These results indicate that the time-responsive dual-peptide delivery pep@MSNs-RA provided a niche-like native ECM, which enhanced stem cell growth to form mature bone.

### Biomimetic DDSs

Furthermore, another key strategy developed biomimetic DDSs. Faced with the complexity of the native tissue microenvironment and adverse side effects, biomimicry is receiving increasing attention.^78^ Compared with traditional DDSs, biomimetic materials could imitate the intricate ECM composition and architecture, thus providing a molecular platform to enhance the control over the delivery of therapeutic molecules, bioactive cues and instructive signals.^79,80^ Biomimetic biomaterials with biological and physicomechanical features inspired by the natural ECM were shown to be able to regulate tissue regeneration.^81^ Biomimetic hydrogels, biomimetic micelles, biomimetic liposomes, biomimetic dendrimers, biomimetic polymeric carriers and biomimetic nanostructures were developed.^82,83^ They mimicked the natural ECM to provide a desirable cellular environment to support cell growth and biodegradation.^84^ Furthermore, biomimetic peptide-based, self-assembly hydrogels (RADA16) were used as an intraosseous delivery vehicle for BMP2 into defects within the femoral head model, thus promoting osteoblastic differentiation of BMSCs and bone remodelling.^85^ This was achieved by activating the phosphorylation of SMAD 1/5/8, a well-established upstream indicator of the BMP2 signalling cascade and a precursor to osteoblastic gene transcription.^85^ In addition, considering the benefits of hollow-channel materials to promote vascularization and the advantages of the unique structure in lotus roots, lotus root-like biomimetic materials with parallel multi-channels were fabricated via a modified 3D printing strategy.^86^ Compared with traditional 3D-printed scaffolds, these biomimetic lotus root-like structures could successfully induce blood vessels and new bone tissues to grow into the inner locations of the biomimetic materials and effectively promote bone defect healing. Therefore, these smart scaffolds possessed better angiogenic and osteogenic stimulatory capabilities, with substantial potential for cell delivery and bone regeneration applications.^86^

## Smart biomaterials and constructs to promote dental and periodontal regeneration

Another important application for smart biomaterials and constructs is periodontal regeneration. Periodontitis is a prevalent chronic inflammatory disease and the leading cause of tooth loss.^87,88^ Periodontitis is initiated by microbes; it leads to periodontal supporting tissue destruction and eventually tooth loss, which significantly affects the quality of life and is a worldwide health burden.^89^ However, the regeneration of periodontal tissues remains a major challenge due to the complexity of the hierarchical architecture of the periodontal ligament (PDL) interspersing between the tooth root cementum and the anchoring bone, oral hygiene, oral fluids, bacterial infection, etc. In addition, the damaged periodontal tissues have a poor innate ability to regenerate. Therefore, there is an urgent need to develop smart materials that can respond to the infection, stimulate the innate regenerative capability and mimic the original architecture and function of the periodontium.^90^

Previous studies have focused on alveolar bone remodelling (including bone formation and resorption) in the bone microenvironment to promote the osteogenic differentiation of stem cells or decrease the differentiation and activity of osteoclasts, with the aim of enhancing alveolar bone regeneration.^91–96^ Other studies attempted to activate the host innate ability, recruit host stem cells to the defect and promote their differentiation, and create an advantageous microenvironment for reducing inflammation and accelerating the bone-healing process.^97–99^ More recent studies focused on the unique structure of the periodontium, with efforts to imitate the structure by designing multiphasic scaffolds to regenerate the PDL and the entire periodontium.^100–106^

Efforts were made to stimulate and harness the self-repair capacity for periodontal regeneration using biomaterials and biomolecules, such as growth factors.^107^ Guided tissue regeneration (GTR) was the first generation of innate regeneration, which employed a membrane around the defect as a barrier to smartly prevent epithelial and fibroblast growth into the defect. This method helped maintain the space for bone and PDL regeneration, while recruiting host progenitor/stem cells to the wound.^108^ As infection was a main reason for periodontitis and clinical failure in regeneration, functional membranes were designed to respond to germs with antibacterial and anti-inflammation properties.^97,98^ A wide range of antimicrobials, including amoxicillin and metronidazole, were added into the polymer membranes for periodontal repairs.^97,110–112^ For example, nanocomposite polycaprolactone-based membranes with amoxicillin were synthesized, which simultaneously provided antibacterial and osteoconductive properties.^113^ Other alternative agents, such as chitosan, could also be incorporated into the membrane.^114^ Moreover, chitosan could entrap biomolecules by crosslinking and ionic complexation and was thus used as a drug and growth factor carrier in periodontal regeneration.^114–116^ Furthermore, collagen was combined with different crosslinking agents, biomaterials and cytokines to improve the mechanical properties and bioactivities, for example, by forming immune-responsive collagen membranes in GTR.^98^ GTR yielded beneficial clinical outcomes in periodontal regeneration and was applied in clinical treatments.

There were reports of excessive immune responses to periodontal bacteria,^117^ and macrophages played a basic role in the onset and progression of periodontitis.^117,118^ Therefore, smart materials were designed to target the macrophages in a harnessing manner to control the inflammation and enhance the innate regeneration. A previous study focused on the immunomodulatory effect of stem cells from human exfoliated deciduous teeth (SHEDs) on the macrophage phenotypic switch.^119^ It was found that SHEDs could promote the classically activated M1 (promote inflammation) to alternative M2 (inhibit inflammation), thereby decreasing the local inflammation and enhancing periodontal regeneration.^119^ Another study investigated microspheres self-assembled with heparin-modified gelatin nanofibres and showed that the microspheres acted as an osteoimmunomodulatory scaffold, producing a pro-regenerative microenvironment for periodontal regeneration under DM.^99^

Furthermore, the functionally oriented PDL inserting into cementum and alveolar bone, as well as the interplay of these three types of tissues, pose a major challenge for tissue engineering. One approach to address this challenge was the development of multiphasic and multi-layered scaffolds that mimic the structure and property of the different tissues.^102^ Bilayered, biphasic, hybrid, triphasic scaffolds were developed to utilize two or three different architectures, materials and composites for periodontal regeneration.^100,101,103,104,106,107,120^ Other technologies, including cell sheets, 3D printing, electrospinning and electrospray, were used in developing multiphasic scaffolds for periodontal regeneration.^100,101,103,104,106,107,120^ In particular, bilayered poly (lactic‐*co*‐glycolic acid) (PLGA)/calcium phosphate constructs were applied in periodontal defects in beagle dogs.^107^ A biphasic scaffold composed of a bone compartment (a fused deposition modelling scaffold) and a PDL compartment (an electrospun membrane combined with PDLSC sheet) were designed for simultaneous PDL and alveolar bone regeneration.^107^ In addition, a tri-layered nanocomposite hydrogel scaffold was developed. To fully mimic the structure of the periodontium, the scaffold consisted of the alveolar bone phase of chitin-PLGA/nanobioactive glass ceramic (nBGC)/platelet-rich plasma derived growth factors, as well as the PDL phase of chitin-PLGA/fibroblast growth factor and the cementum phase of chitin‐PLGA/nBGC/cementum protein 1.^121^ The multiphasic scaffolds were promising in inducing cells for cementogenic differentiation onto tooth root surfaces, encouraging PDL alignment to the cementum and bone, and providing osteogenesis signals for bone formation.^121^ However, the natural structure and physical and chemical properties of the periodontium remained difficult to mimic. The adaptation of the scaffold on the interface between the different types of tissues remained poor, and the material’s resorption rate and the tissue regeneration rate remained difficult to match. Therefore, further studies are required to improve the multiphasic/multi-layered scaffolds, optimize cell and growth factor delivery and their tailored interactions, and undertake animal studies on periodontal regeneration to translate the smart tissue engineering constructs from the laboratory to the clinic.

## Smart dental resins that respond to pH to protect tooth structures

One important smart stimuli-responsive approach in dentistry employs materials that can respond to pH to protect the tooth structures. Dental caries is prevalent worldwide, is one of the most common bacterial infections in humans and represents a heavy financial burden.^122,123^ The basic mechanism of caries is demineralization due to attack by acid produced by bacteria.^123–125^ Oral acidogenic bacteria ferment carbohydrates and produce organic acids, including lactic, formic, acetic and propionic acids.^123^ Following a sucrose rinse, the local plaque pH can decrease to 4.5 or 4.^126^ Regarding the protection of tooth structures, there is a critical pH below which demineralization dominates, causing mineral loss.^127^ For most individuals, this critical pH is approximately 5.5.^126^ The Stephan Curve shows that the plaque pH, following a glucose rinse, remains in the cariogenic area of close to pH 4 for approximately 30 min and then increases back to a safe pH of >5.5 after the bacteria have completed their metabolization of the glucose and the saliva has buffered the acid.^126^ Therefore, it would be highly desirable to develop a smart resin for tooth cavity restorations that responds to pH by releasing high levels of calcium and phosphate ions and neutralizing the acids at a low pH when these ions are most needed for caries inhibition. Moreover, when the pH is close to neutral, the smart resin would have little release to preserve the ion reservoir.

**Fig. 4:**
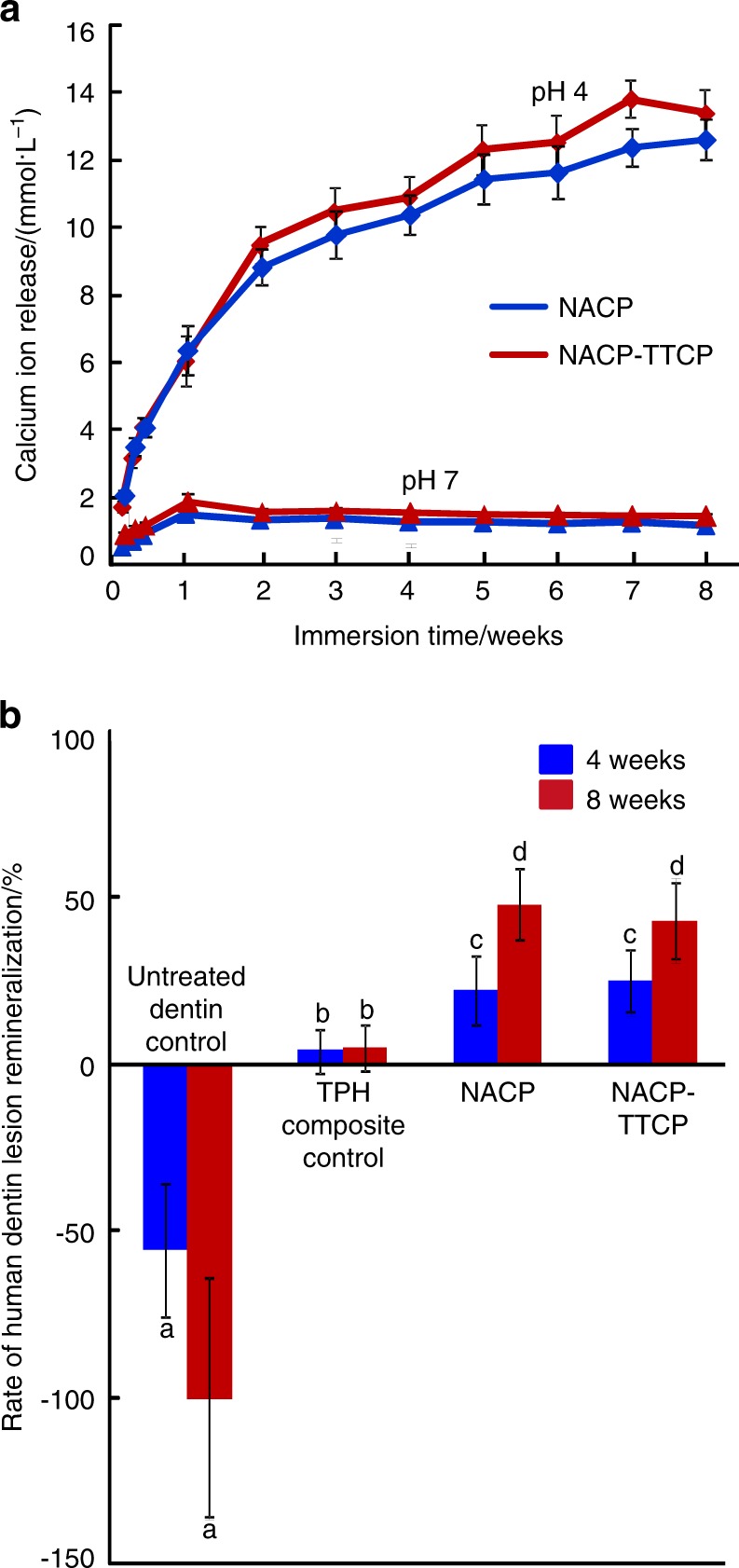
Smart dental composites for caries inhibition. **a** Composites with calcium (Ca) ion release. The phosphate (P) ion release had a similar trend. Ion release substantially increased at a cariogenic pH 4 when these ions were most needed to combat caries. There was little release at a neutral pH to preserve the ion reservoir. **b** Remineralization. Dentin lesions without composite had substantial mineral loss. Dentin lesions restored with commercial composite had little remineralization. The smart composites caused successful remineralization. (Adapted from ref. ^128^, with permission.)

Dental resin composites were developed that exhibited substantial calcium ion release at a cariogenic pH 4 and limited ion release at pH 7 (Fig. 4a).^128^ Phosphate ion release showed a similar trend.^128^ The two composites in Fig. 4 consisted of nanoparticles of amorphous calcium phosphate (NACP) with a mean particle size of approximately 116 nm, which were synthesized using a spray-drying technique,^129^ in addition to tetracalcium phosphate particles with a median of 0.8 μm. These composites were tested in dentin caries restorations in vitro, and the constructs were subjected to a cyclic demineralization and remineralization regimen for 4–8 weeks.^128^ Transverse microradiography was performed to measure the mineral content in dentin, and the percentage of mineral change was used to determine the remineralization in Fig. 4b (mean ± SD; *n* = 15). Due to the cyclic regimen of 23 h in pH 4 and 1 h in pH 7 daily, the dentin lesion without a composite had mineral loss at 4 weeks (−55.7 ± 20.3)% and 8 weeks (−100.66 ± 35.8)%. A commercial composite (TPH) caused no remineralization as expected. In contrast, the smart composites achieved approximately 50% remineralization for the dentin lesions in 8 weeks.^128^ In addition to dentin, a separate study demonstrated enamel remineralization by smart composite that was fourfold the remineralization achieved by a commercial fluoride control.^130^

**Fig. 5:**
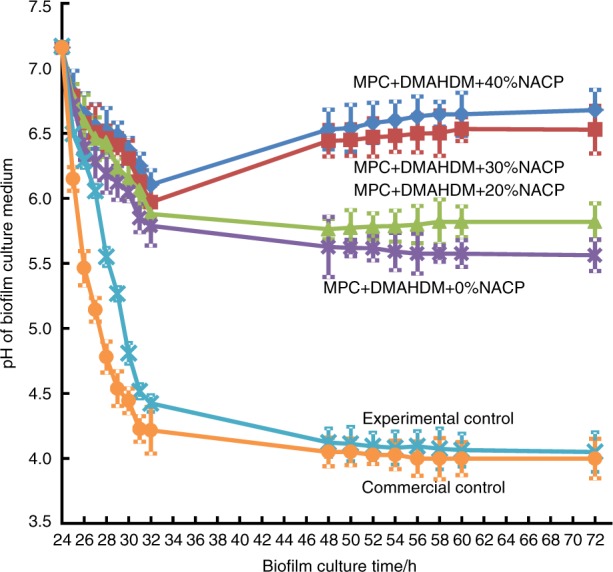
Effect of smart resins on neutralizing biofilm acids and increasing the pH to avoid tooth decay. The adhesive resin contained MPC, DMAHDM and NACP from 0 to 40%. A dental plaque microcosm biofilm model was used with human saliva as inoculum. The pH of the biofilm medium with resin that contained 30 and 40% NACP was at pH 6 or greater. The pH of the biofilm medium with commercial adhesive was cariogenic at approximately pH 4, which could demineralize the tooth structures. (Adapted from ref. ^132^, with permission.)

Moreover, a human in situ study was performed in which 25 volunteers wore palatal devices that contained enamel slabs with cavities restored using a smart NACP composite or a control composite, where the restorations were covered with biofilms in vivo fed with sucrose to produce acids.^131^ The enamel mineral loss at the restoration margins around the smart composite was only 1/3 of the mineral loss around the control composite.^131^ The mechanism of caries inhibition for the smart resins was demonstrated in a recent study, in which the pH of the biofilm medium was monitored.^131^ In this study, a smart adhesive resin contained NACP, antibacterial dimethylaminohexadecyl methacrylate (DMAHDM) and protein-repellent 2-methacryloyloxyethyl phosphorylcholine (MPC). As shown in Fig. 5,^132^ the oral biofilm pH decreased with increasing time due to acid production by the biofilms and reached a cariogenic pH 4.0 for the commercial control adhesive, pH 4.1 for the experimental control (no DMAHDM, no MPC and no NACP) and pH 5.6 for the adhesive resin with MPC + DMAHDM + 0% NACP. For the smart resins that contained NACP, the biofilm pH increased with an increasing NACP mass fraction and reached pH 6 with 30% NACP and pH 6.5 with 40% NACP at 72 h.^132^ These pH values are well within the safe zone of >5.5 to suppress caries formation. Further studies are required to investigate smart materials that respond to pH for dental applications, as well as for bone repair and regeneration, for example, to release calcium and phosphate ions triggered by overactive osteoclasts to potentially neutralize acids and suppress osteoporosis.

## Smart pH-sensitive materials to selectively inhibit acid-producing bacteria

**Fig. 6:**
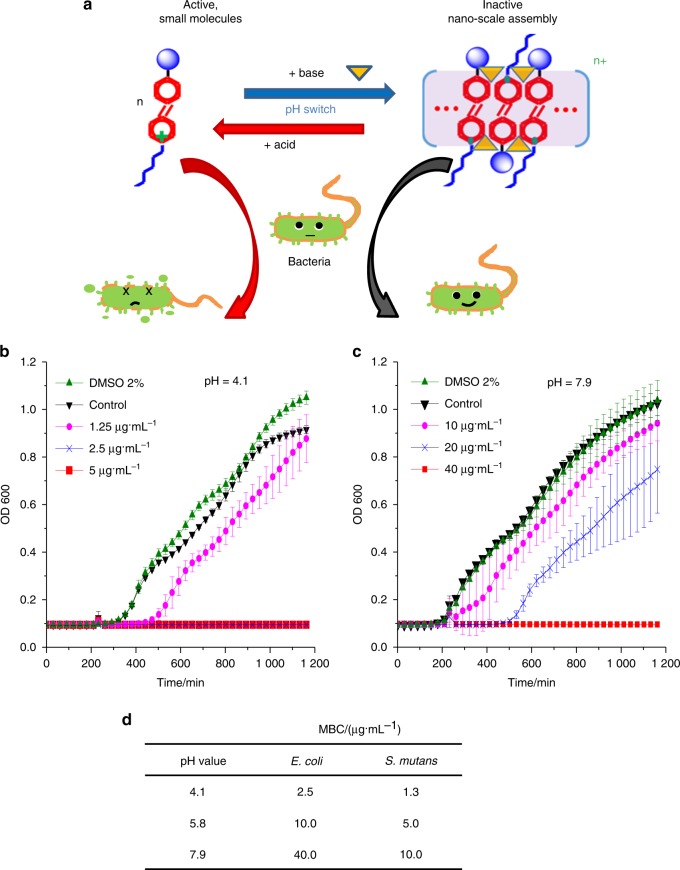
Smart pH-sensitive material for selective inhibition of acid-producing bacteria. **a** pH-sensitive, reversible spectroscopic properties and assembly behaviour of Azo-QPS-C16. The schematic shows the assembly and disassembly between Azo-QPS-C16 and its nano-sized aggregates, mediated by pH switch or addition of base/acid, for reversible control of the antibacterial activity. Acid-enhanced the antibacterial behaviours of Azo-QPS-C16, with *E. coli* growth curves after treatment with different concentrations of Azo-QPS-C16 at **b** pH 4.1 and **c** pH 7.9. **d** MBC values of Azo-QPS-C16 against *E. coli* and *S. mutans* at different pH values. (Adapted from ref. ^135^, with permission.)

In addition to the pH-triggered release of cavity-fighting ions, another type of smart biomaterials possesses on-demand antimicrobial capability and can produce stimuli-responsive antibacterial activity on-site. They enable the treatment of infection locally with less antimicrobial agent, which can help reduce antimicrobial drug resistance.^133,134^ Previous studies developed optically controlled antibacterial function to improve the efficacy of drugs via light irradiation.^133,134^ In addition, the antibacterial function of a supramolecular complex was reversibly switched on band off through the assembly and disassembly of a cationic poly(phenylene vinylene) derivative.^134^ Recently, pH-sensitive quaternary pyridinium salts (QPS) were developed, for which the antibacterial potency is boosted by low pH and can be controlled by varying the pH between 4 and 8.^135^ This material can selectively suppress the growth of acidogenic bacteria within a multispecies biofilm. This molecule, (E)-1-hexadecyl-4-((4-(methacryloyloxy)phenyl)diazenyl)-pyridinium bromide (termed Azo-QPS-C16), can adjust its antibacterial potency within pH 4–8. Therefore, the biocidal activity of Azo-QPS-C16 can have localized triggering by acidic metabolic products of bacteria, which, in turn, leads to the killing of these bacteria. As shown in Fig. 6a,^135^ the antibacterial function is switched on via an acidic environment through the disassembly of the agglomerates, thus releasing Azo-QPS-C16. This increases the number of active antimicrobial sites, which is termed “antibacterial on”. In contrast, in a neutral or mildly basic environment, this process is reversed, which is termed “antibacterial off”.

Figure 6b, c plot the *Escherichia coli* (*E. coli*) growth curves after treatment with pH 4.1 and pH 7.9 buffers.^135^ At pH 4.1, a concentration of 2.5 µg/mL of Azo-QPS-C16 was sufficient to completely inhibit *E. coli* growth. In sharp contrast, at pH 7.9, 40 µg/mL of Azo-QPS-C16 was required to completely inhibit *E. coli* growth. The bacteria were subsequently treated with Azo-QPS-C16 for 30 min using a 2-fold serial dilution. The effect of pH on the antibacterial potency of Azo-QPS-C16 in terms of the minimum bactericidal concentration (MBC) was then measured by inoculating cultures onto a Lysogeny broth agar plate. As shown in Fig. 6d, the MBC increased with increasing pH. From pH 4.1 to 7.9, the MBC for *E. coli* was increased by 16-fold. A similar pH-sensitive antibacterial function was shown for the cariogenic *Streptococcus mutans* (*S. mutans*) (Fig. 6d, third column). These results demonstrate that the smart pH-sensitive materials can selectively inhibit acid-producing bacteria, thereby providing protection against infection and erosion via targeted treatments against acid-producing bacteria. These smart materials are promising for applications not only in combating tooth caries but also in inhibiting the selected pathogens for orthopaedic implants and tissue engineering scaffolds.

## Smart resins to modulate biofilm species towards a healthy composition

Another class of smart materials aims to modulate the oral biofilm composition by suppressing cariogenic and pathological species and promoting non-cariogenic and healthy species. Quaternary ammonium methacrylates (QAMs) are cationic compounds with a broad spectrum of antimicrobial effects.^136–139^ The antimicrobial mechanism of QAMs occurs via the disruption of bacterial membranes.^136–139^ Compared to release-based biomaterials, QAMs can be co-polymerized with the resin matrix to anchor itself into the polymer network with prolonged functions. The first QAM used in dental resin was 12-methacryloyloxy dodecyl pyridinium bromide (MDPB), which exerted potent antibacterial effects against biofilm growth on resins.^137–139^ Several other QAMs were subsequently developed, including quaternary ammonium polyethylenimine,^140^ methacryloxylethyl cetyl dimethyl ammonium chloride (DMAE-CB),^141^ dimethylaminododecyl methacrylate (DMADDM),^142^ and DMAHDM.^143,144^

**Fig. 7:**
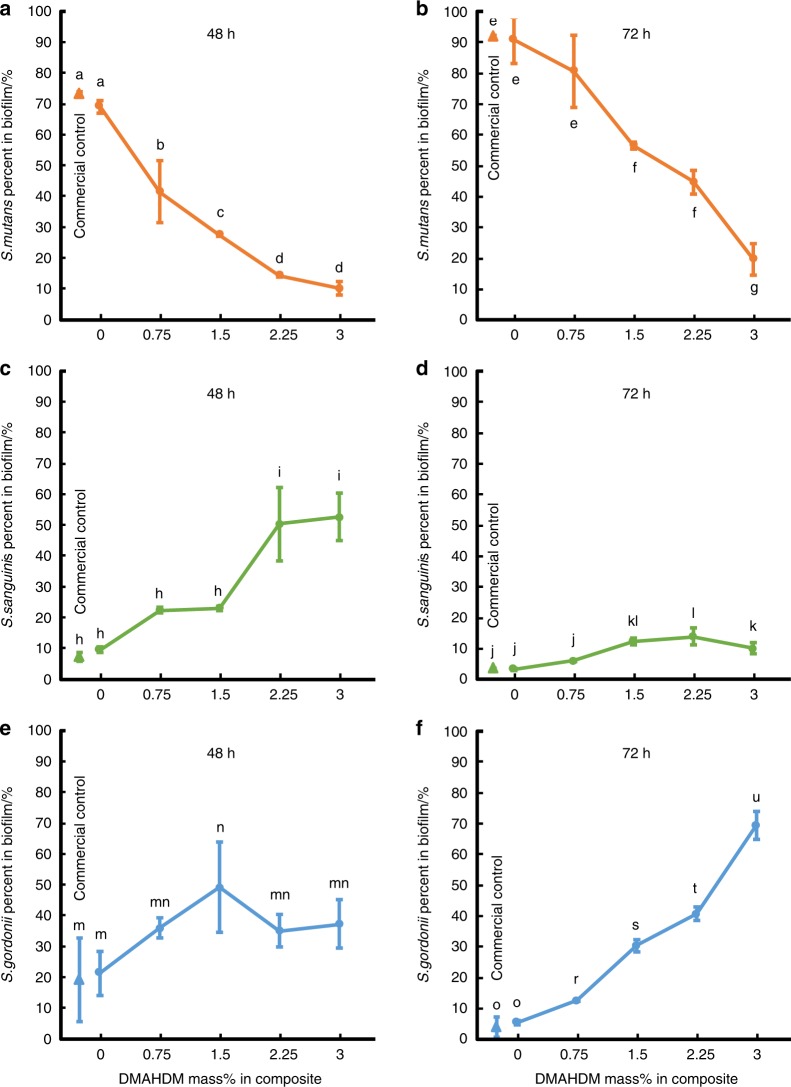
Smart composite to shift species in biofilms towards a healthy composition. Three-species biofilms (*S. mutans*, *S. sanguinis* and *S. gordonii*) were grown on composites for 48 and 72 h. The commercial composite was Heliomolar (Ivoclar). The DMAHDM wt% was varied in the experimental composite from 0 to 3%. The cariogenic *S. mutans* had overwhelming proportions for the commercial control and 0% DMAHDM group. With increasing DMAHDM wt%, the proportion of *S. mutans* sharply decreased, whereas *S. sanguinis* or *S. gordonii* achieved a predominant proportion. Values with dissimilar letters are significantly different (*P* < 0.05). (Adapted from ref. ^146^, with permission.)

The dental plaque contains multispecies microbial communities. Variation in the oral environment can trigger a species change in the microflora. Frequent sugar intake can promote the acidogenic and aciduric species at the expense of the healthy and less aciduric residents, thereby shifting the dental plaque towards a cariogenic composition.^145^ Therefore, it would be highly desirable to develop smart resins to modulate the biofilms from a cariogenic state and shift it towards a non-cariogenic microbial community. This important direction of research has only recently been initiated and is in its early stage. A literature search indicated only one report on the use of QAM to modulate oral biofilms.^145^ This study demonstrated that a resin containing DMADDM was able to suppress the cariogenic *S. mutans* and promote the growth of non-cariogenic species.^145^ In addition to this published study, another study (in press) on a DMAHDM composite showed that it suppressed cariogenic species and promoted non-cariogenic species in oral biofilms.^146^ A biofilm model that consisted of *S. mutans*, *Streptococcus sanguinis* (*S. sanguinis*) *and Streptococcus gordonii* (*S. gordonii*) was tested by growing biofilms on composites for 48 and 72 h. As shown in Fig. 7, at 48 h, the commercial control and the experimental composite with 0% DMAHDM had *S. mutans* proportions of 73% and 69%, respectively. The *S. mutans* proportion decreased with an increasing DMAHDM mass fraction, accounting for 15% in the 2.25% DMHADM group and 10% in the 3% DMHADM group. At 72 h, *S. mutans* in the commercial control and 0% DMAHDM group reached overwhelming proportions of 92% and 91%, respectively. In contrast, the *S. mutans* proportion decreased to 20% in the biofilm on the composite with 3% DMHADM. Moreover, the proportions of non-cariogenic *S. sanguinis* and *S. gordonii* increased with an increasing DMAHDM content. Therefore, the DMAHDM composite has the potential to modulate the biofilm composition shift from a cariogenic state to a non-cariogenic state. Further studies are required to develop smart biomaterials to suppress pathogenic species and promote healthy species in biofilms not only for dentistry but also bone regeneration and tissue engineering applications. For example, it would be beneficial to develop smart constructs for periodontal regeneration that can suppress periodontal pathogens and promote healthy species to combat the progression of periodontal diseases and infections, which warrants further studies.

## Smart tailoring of alkyl chain length in QAM to avoid drug resistance

While it is beneficial for dental resins to be able to suppress bacteria and modulate biofilms, it is important that they do not induce drug resistance. Frequent use of antibiotics may lead to a selection of resistant strains or the emergence of acquired resistance to these sensitive strains.^147^ Bacterial drug resistance is a significant and increasing concern.^147^ Previous studies have indicated that *Staphylococcus aureus* (a common cause of skin infections, respiratory infections and food poisoning), *Serratia marcescens* (involved in hospital-acquired infections, such as urinary tract infections and wound infections) and *E. coli* (occasionally responsible for product recalls due to food contamination) could acquire resistance against quaternary ammonium compounds.^148,149^

**Fig. 8:**
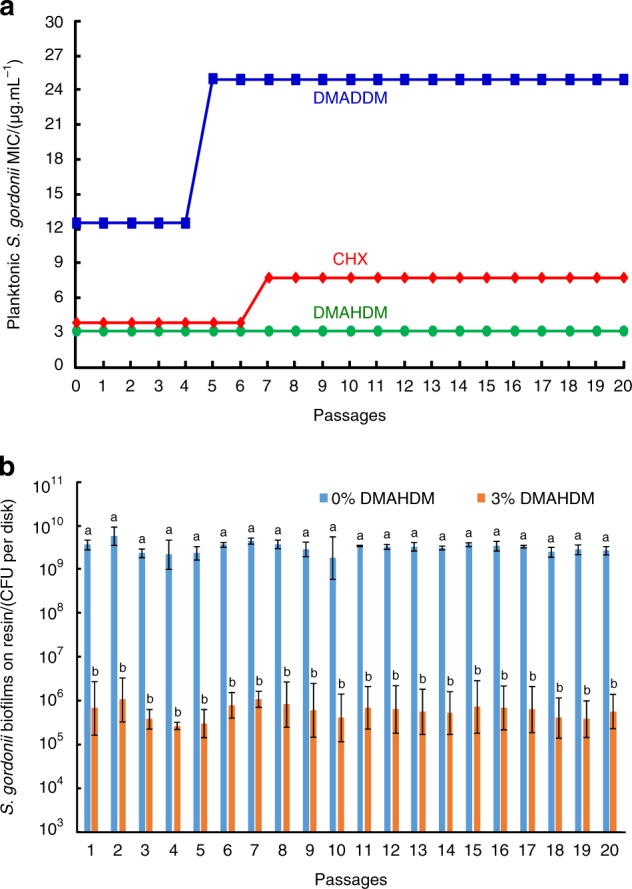
Smart inhibition of bacteria without inducing drug resistance. **a** Minimal inhibitory concentration (MIC). Twenty passages were tested, and each passage used the surviving bacteria from the previous passage as inoculum. A twofold increase in MIC occurred for DMADDM and CHX, indicating drug resistance. However, DMAHDM had no drug resistance. **b** From 1 to 20 passages, *S. gordonii* biofilm CFU on DMAHDM resin maintained a 4 log reduction over that without DMAHDM, showing the same sensitivity with no drug resistance. In **b**, bars with dissimilar letters are significantly different from each other (*P* < 0.05). (Adapted from ref. ^146^, with permission.)

Research on the possibility of oral bacteria developing resistance to QAMs is only in the early stage. A literature search indicated that to date, there are only three publications on this important topic.^150–152^ One study showed that after serial exposures to cationic biocides, *S. mutans* and *Enterococcus faecalis* (*E. faecalis*, which is closely associated with apical periodontitis) did not exhibit resistance to MDPB after repeated exposures, while *E. faecalis* developed drug resistance to chlorhexidine (CHX).^150^ Another study showed that DMAHDM did not induce drug resistance, while DMADDM and CHX induced drug resistance for *S. gordonii* by testing repeated exposures for 10 passages.^151^ Another study investigated the drug resistance against DMAHDM by further increasing the repeated exposures to 20 passages, with each passage using the exposed and survived bacteria as inoculum for the subsequent passage.^152^ As shown in Fig. 8a, for DMADDM, the minimal inhibitory concentration (MIC) of planktonic *S. gordonii* increased from 12.5 to 25 µg·mL^-1^ at passage 5 and remained at 25 µg/mL until passage 20. For CHX, the MIC increased from 3.9 to 7.8 µg·mL^-1^ at passage 7 and then remained constant until passage 20. In contrast, the MIC remained constant at 3.125 µg/mL for DMAHDM. Therefore, DMADDM and CHX induced bacterial resistance in *S. gordonii*; however, DMAHDM did not induce drug resistance. In Fig. 8b, for each passage, the addition of DMAHDM into the resin substantially decreased the biofilm biomass, with nearly 4 logs of reduction in colony-forming units (CFUs). Furthermore, there was no change in the extent of the CFU reduction after passaging the survived bacteria with repeated exposures to the DMAHDM resin. Therefore, the biofilm remained highly and similarly susceptible to the DMAHDM resin from passages 1 to 20, showing no sign of drug resistance.^152^

DMADDM caused a mild drug resistance in *S. gordonii*, while DMAHDM did not induce drug resistance. Regarding the mechanism, DMADDM and DMAHDM are membrane-active antibacterial agents that interact with bacteria membranes. The quaternary ammonium antibacterial mode of action is related to the alkyl chain length (CL). Longer chains can more readily penetrate bacterial cells, similar to a needle bursting a balloon. DMAHDM with a longer CL of 16 exhibited a stronger antibacterial potency than DMADDM with a CL of 12.^153^ The results that *S. gordonii* had drug resistance to DMADDM, but not to DMAHDM, indicate that the drug-resistant ability in bacteria is related to CL. The way that bacteria develops resistance may be to congeal or thicken the bacterial membrane and thereby resist the destruction of biocide alkyl action and prevent membrane leakage. It is likely that DMAHDM with longer alkyl chains could penetrate the hydrophobic bacterial membrane to cause bacteria lysis. Therefore, it appears to be more difficult for bacteria to acquire resistance against longer chains. These results indicate that longer alkyl chains (1) have stronger antimicrobial potency and (2) can make it more difficult for bacteria to develop drug resistance.^152^ In addition to the CL, other parameters, such as the quaternary amine charge density on resins^143,153^ and multiple modes of action by combining multiple agents,^154^ may also provide ways to suppress the ability of bacteria to acquire drug resistance and warrant further investigation. Therefore, intelligently tailoring material parameters may enable the design of smart dental resins to exert antibacterial functions without inducing drug resistance. Furthermore, the smart design of antibacterial dental materials may have applicability for bone engineering constructs to possess antibacterial activity, without inducing drug resistance, to combat infections in wounds while regenerating bone tissues.

## Summary

This article reviewed recent developments in novel smart biomaterials for hard tissue engineering and regeneration. The cutting-edge research included smart scaffolds and stem cell constructs for bone tissue engineering, smart DDSs, smart constructs for periodontal regeneration, smart dental resins that respond to pH to protect tooth structures, smart pH-sensitive materials to selectively inhibit acid-producing bacteria, smart resins to modulate biofilm species towards a healthy composition and smart tailoring of materials to avoid drug resistance. Smart biomaterials/drug delivery and stem cell methods for bone tissue engineering are also applicable to dentistry, including maxillofacial, periodontal and pulp regenerations. Moreover, some of the smart approaches being developed in the dental field are also applicable to bone tissue engineering. For example, smart materials for tooth remineralization may benefit treatments for osteoporosis. In addition, smart dental materials, which can suppress bacteria and modulate biofilm species without inducing drug resistance in oral bacteria, may have applicability to bone engineering with infection control in wound sites. One disadvantage is that there has been only a relatively small number of systems of smart biomaterials reported to date, thus limiting the choice of available compositions. Therefore, future efforts are required to develop more novel compositions with improved and better-controlled smartness and to translate the in vitro smartness properties to effective in vivo tissue repair and regeneration in animal models. Overall, the new generation of smart biomaterials provides exciting potential and is promising to substantially enhance hard tissue engineering and regenerative medicine efficacy.
